# Mapping of Cervical and Upper Mediastinal Lymph Node Recurrence for Guiding Clinical Target Delineation of Postoperative Radiotherapy in Thoracic Esophageal Squamous Cell Carcinoma

**DOI:** 10.3389/fonc.2021.663679

**Published:** 2021-04-26

**Authors:** Yichun Wang, Dongmei Ye, Mei Kang, Liyang Zhu, Mingwei Yang, Jun Jiang, Wanli Xia, Ningning Kang, Xiangcun Chen, Jie Wang, Fan Wang

**Affiliations:** ^1^ Department of Radiation Oncology, The First Affiliated Hospital of Anhui Medical University, Hefei, China; ^2^ Department of Thoracic Surgery, The First Affiliated Hospital of Anhui Medical University, Hefei, China; ^3^ Department of Medical Imaging, The First Affiliated Hospital of Anhui Medical University, Hefei, China

**Keywords:** esophageal neoplasm, adjuvant radiotherapy, lymph nodes, recurrence/analysis, irradiation field

## Abstract

**Background:**

The lower neck and upper mediastinum are the major regions for postoperative radiotherapy (PORT) in thoracic esophageal squamous cell carcinoma (TESCC). However, there is no uniform standard regarding the delineation of nodal clinical target volume (CTVnd). This study aimed to map the recurrent lymph nodes in the cervical and upper mediastinal regions and explore a reasonable CTVnd for PORT in TESCC.

**Methods:**

We retrospectively reviewed patients in our hospital with first cervical and/or upper mediastinal lymph node recurrence (LNR) after upfront esophagectomy. All of these recurrent lymph nodes were plotted on template computed tomography (CT) images with reference to surrounding structures. The recurrence frequency at different stations was investigated and the anatomic distribution of recurrent lymph nodes was analyzed.

**Results:**

A total of 119 patients with 215 recurrent lymph nodes were identified. There were 47 (39.5%) patients with cervical LNR and 102 (85.7%) patients with upper mediastinal LNR. The high-risk regions were station 101L/R, station 104L/R, station 106recL/R, station 105 and station 106pre for upper TESCC and station 104L/R, station 106recL/R, station 105, station 106pre and station 106tbL for middle and lower TESCCs. LNR in the external group of station 104L/R was not common, and LNR was not found in the narrow spaces where the trachea was in close contact with the innominate artery, aortic arch and mediastinal pleura. LNR below the level of the cephalic margin of the superior vena cava was also not common for upper TESCC.

**Conclusions:**

The CTVnd of PORT in the cervical and upper mediastinal regions should cover station 101L/R, station 104L/R, station 106recL/R, station 105 and station 106pre for upper TESCC and station 104L/R, station 106recL/R, station 105, station 106pre and station 106tbL for middle and lower TESCCs. Based on our results, we proposed a useful atlas for guiding the delineation of CTVnd in TESCC.

## Introduction

Esophageal carcinoma (EC) is a common malignant tumor of the upper digestive tract. Surgery remains the cornerstone of curative treatment for resectable EC. Unfortunately, EC is commonly found at an advanced stage when surgery alone cannot achieve cure and is associated with a poor survival (1). For patients with advanced diseases, a multidisciplinary treatment strategy should increase their chances of survival. Many trials on adjuvant or neoadjuvant therapy have been conducted in past decades (2). After the success of the ChemoRadiotherapy for Oesophageal Cancer Followed by Surgery Study (CROSS) trial, neoadjuvant chemoradiotherapy has become the standard treatment for locally advanced resectable EC in many countries (3, 4). Although postoperative radiotherapy (PORT) has failed to improve survival in EC according to most previous randomized controlled trials, these trials have been criticized for many limitations, and an increasing number of studies have proved that postoperative radiotherapy/chemoradiotherapy can improve the survival of patients with poor prognosis after upfront esophagectomy (5). Therefore, PORT with or without chemotherapy is still a choice for EC in clinical practice, especially for patients with pathologically upstaged clinical early stage EC or patients with locally advanced EC who receive upfront surgery.

Many clinical target volumes have been used for PORT in EC in past decades, ranging small volumes consisting of the primary tumor bed only to large volumes consisting of the lower neck, total mediastinum and the upper celiac area (5). An appropriate irradiation field may not only improve disease control but also decrease radiotherapy-related complications. Due to the characteristics of lymphatic drainage, surgical procedure and recurrence patterns after esophagectomy (6–8), it has been found that the lower neck, upper mediastinum and paraaortic area are high-risk regions for lymph node recurrence (LNR) in thoracic EC (TEC). Therefore, it is recommended that the lower cervical and upper mediastinal regions should be included in the nodal clinical target volume (CTVnd) for PORT in all TECs, while the celiac region should also be included in the CTVnd in TECs with a high-risk factor for celiac recurrence (5, 6, 9).

The delineation of CTVnd in the lower cervical region has been proposed in TEC without surgery (10, 11). However, there is no uniform delineation of CTVnd in the cervical and upper mediastinal regions for PORT in TEC. Many important stations of lymph nodes (LNs) have been proposed for irradiation, while the precise sites of recurrent LNs in these stations are not clear (6–8). Here, we reviewed the data of patients in our hospital with thoracic esophageal squamous cell carcinoma (TESCC) who received R0 esophagectomy without perioperative antitumor therapy, aiming to map all of the nodal recurrence sites in the cervical and upper mediastinal regions on template computed tomography (CT) images and to identify a more precise CTVnd for PORT in TESCC.

## Materials and Methods

### Study Populations

From June 2012 to June 2020, patients in our hospital diagnosed with pathological stage T1-4aN0-3M0 (the 8th edition of AJCC classification) TESCC were reviewed. After systematic screening, all included patients met the following criteria: 1) patients who received R0 esophagectomy without perioperative antitumor therapies before recurrence; 2) patients with two-field or three-field lymphadenectomy; and 3) patients with cervical and/or upper mediastinal LNR at the time of first recurrence. We excluded patients if they: 1) had unclear pathological records; 2) had double or multiple primary cancers; 3) had uncertain recurrence sites (imaging data were not available); and 4) had anastomotic recurrence or hematologic metastasis at the time of first recurrence.

### Regional Lymph Node Division

The names, numbers and atlas of regional node divisions of the neck and upper mediastinum are based on the Japanese Classification of Esophageal Cancer (12, 13). The classification standards are as follows: 1) cervical LNs include station 101L/R (cervical paraesophageal LNs), station 102 L/R (deep cervical LNs), station 103L/R (peripharyngeal LNs) and station 104 L/R (supraclavicular LNs); and 2) upper mediastinal LNs include station 105 (upper thoracic paraesophageal LNs), station 106 (106rec L/R: recurrent nerve LNs; station 106pre: pretracheal LNs; station 106tbL/R: tracheobronchial LNs), station 113 (ligamentum arteriosum LNs) and station 114 (anterior mediastinal LNs).

### Follow-Up

Follow-up after surgery was conducted every 2-3 months for the first 6 months, every 3-6 months thereafter within the first 2 years and every 6-12 months after 2 years. Chest CT, abdominal and cervical ultrasound or CT was implemented for re-examinations. When a suspicious LN was found by ultrasound, CT, magnetic resonance or PET/CT was performed.

### Diagnosis of LNR

CT, occasionally magnetic resonance or PET/CT was used for the diagnosis of cervical and mediastinal LNR. Fine needle aspiration cytology was also used when necessary. LNs with a short diameter greater than 10 mm (5 mm in the tracheoesophageal groove) and LNs with fusion, necrosis or increasing size during follow-up were considered to represent LNR, and LNs with a HUVmax value greater than 2.4 on PET/CT images were considered to represent LNR.

### Mapping of Recurrent LNs on Template CT

Due to the great differences in anastomoses and the mediastinal stomach after surgery among patients, we selected a 63-year-old woman with newly diagnosed lower TESCC in the real world as a standard patient. She underwent contrast-enhanced CT scanning from the skull base to 5.00 cm below the tracheal carina with both hands holding the contralateral elbow and raised to contact the forehead. There was no identified enlarged LN and no obvious abnormality in her neck or mediastinum. Template CT images with a 1.25-mm-thick section were imported into the Pinnacle 3 treatment planning system (version 9.8.0.6007; Philips Medical Systems, Fitchburg, WI, USA) for analyses.

All recurrent LNs were transferred to the template CT by two experienced radiation oncologists and an experienced radiologist. The surrounding structures, including the trachea, esophagus, thyroid, blood vessels, muscles and bones, were referred to determine the corresponding anatomic positions. Every recurrent LN was plotted with a diameter of 4.00 mm according to their geometric center. The geometric center of each mixed LN that was distinguishable was plotted in its respective location. Otherwise, only a geometric center was plotted. To better stimulate the primary LNR on the continuous cross-sectional CT images, we also used spheres with a diameter of 1.00 cm to represent all of the primary LNRs. Therefore, a nodal target volume (TVnd) was expanded outward by 3.00 mm and expanded 5.00 mm on the upper and lower bounds for each plotted LN.

### Statistical Analysis

The SPSS statistical software package (version 26.0 for Windows, IBM SPSS, Armonk, NY, USA) was used for statistical analysis. For categorical variables, the Chi-square test was used. A value of P <0.05 was used as the significance threshold.

## Results

### Characteristics of Included Patients

After systematic screening, there were a total of 119 patients included in our analyses. The median interval time from surgery to the first recurrence was 9.0 (1.0 to 77.0) months. LNR in 112 (94.1%), 2 (1.7%) and 5 (4.2%) patients were diagnosed by CT, magnetic resonance and PET/CT, respectively. General information for these patients is shown in **Table 1**. The median age and number of resected LNs were 65.0 (46.0 to 82.0) years old and 15 (4 to 45) respectively. A total of 93 (78.2%) patients had middle TESCC and 107 (89.9%) patients underwent two-field lymphadenectomy. There were 47 (39.5%) patients with cervical LNR and 102 (85.7%) patients with upper mediastinal LNR. There was no significant difference in the upper mediastinal LNR among the different parameters. A higher nodal stage, vessel invasion and perineural invasion in the patients were associated with a high cervical LNR (P = 0.022, 0.026, and 0.002, respectively).

**Table 1:** Clinicopathological characteristics of patients.

Parameters	Number(%)	Cervical LNR		Upper mediastinal LNR	
		Number (%)	P value	Number (%)	P value
Gender			0.640		1.000
Male	91 (76.5)	37 (40.7)		78 (85.7)	
Female	28 (23.5)	10 (35.7)		24 (85.7)	
Age (years)			0.275		0.369
< 65	58 (48.7)	20 (34.5)		48 (82.8)	
≥65	61 (51.3)	27 (44.3)		54 (88.5)	
Location			0.587		0.812
Upper	10 (8.4)	3 (30.0)		9 (90.0)	
Middle	93 (78.2)	39 (41.9)		80 (86.0)	
Lower	16 (13.4)	5 (31.3)		13 (81.3)	
T stage			0.140		0.331
T1	12 (10.1)	3 (25.0)		10 (83.3)	
T2	30 (25.2)	14 (46.7)		23 (76.7)	
T3	72 (60.5)	26 (36.1)		64 (88.9)	
T4a	5 (4.2)	4 (80.0)		4 (80.0)	
Nodal stage			0.022		0.240
N0	61 (51.3)	16 (26.2)		56 (91.8)	
N1	30 (25.2)	15 (50.0)		23 (76.7)	
N2	18 (15.1)	10 (55.6)		15 (83.3)	
N3	10 (8.4)	6 (60.0)		8 (80.0)	
Differentiation			0.081		0.719
Poor	43 (36.1)	21 (48.8)		36 (83.7)	
Moderate	73 (61.2)	26 (35.6)		63 (86.3)	
Well	3 (2.5)	0 (0.0)		3 (100.0)	
Lymphadenectomy			0.163		0.263
Two-field	107 (89.9)	45 (42.1)		93 (86.9)	
Three-field	12 (10.1)	2 (16.7)		9 (75.0)	
Resected LNs			0.733		0.708
<15	58 (48.7)	22 (37.9)		49 (84.5)	
≥15	61 (51,3)	25 (41.0)		53 (86.9)	
Vessel invasion			0.026		0.102
Yes	30 (25.2)	17 (56.7)		23 (76.7)	
No	89 (74.8)	30 (33.7)		79 (88.8)	
Perineural invasion			0.002		0.415
Yes	26 (21.8)	17 (65.4)		21 (80.8)	
No	93 (78.2)	30 (32.3)		81 (87.1)	

### Frequency of LNR in Different Stations

**Figure 1:**
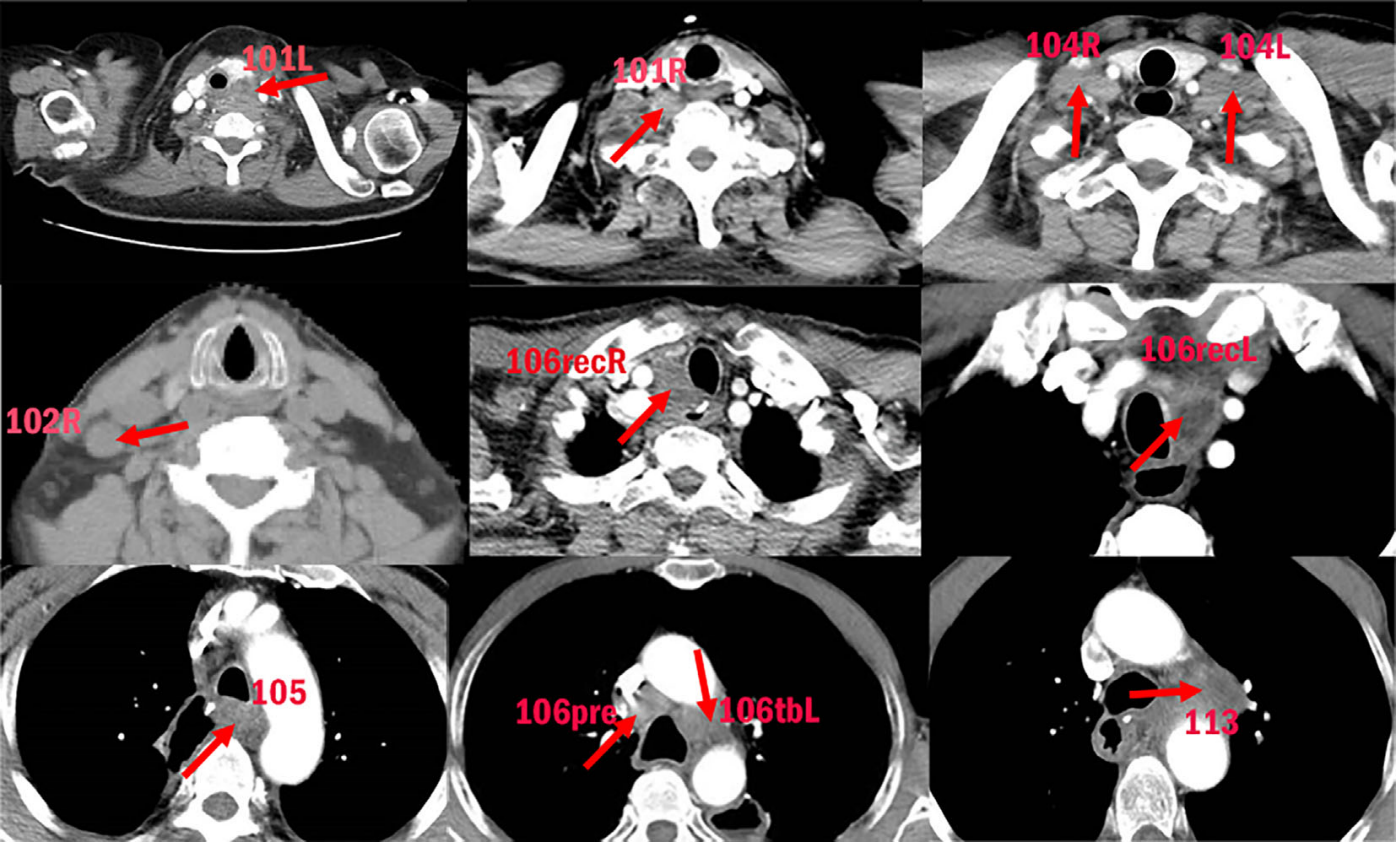
Examples of LNR in different stations.

Examples of LNR at different stations are shown in **Figure 1**. The frequency of LNR and percentage of patients with LNR at different stations are shown in **Table 2**. The high-risk regions of LNR for TESCC were station 106recR (40.3%), station 106recL (39.5%), station 104L (29.0%) and station 104R (18.5%), followed by station 105 (14.3%), station 106pre (11.8%) and station 106tbL (5.9%). LNR was not common at station 101R, station 101L, station 102R, station 102L, station 106tbR, station 113, station 114 or station 103. However, station 101L/R was also a high-risk region while station 106tbL was not a high-risk region of LNR for upper TESCC in this study.

**Table 2:** Frequency of LNR and percentage of patients with LNR at different stations for 119 TESCCs.

Stations	Number of patients (%)			
	Upper (n=10)	Middle (n=93)	Lower (n=16)	Total (n=119)
101R	1 (10.0)	2 (2.2)	0 (0.0)	3 (2.5)
101L	1 (10.0)	2 (2.2)	0 (0.0)	3 (2.5)
102R	0 (0.0)	1 (1.1)	0 (0.0)	1 (0.8)
102L	0 (0.0)	0 (0.0)	0 (0.0)	0 (0.0)
103R	0 (0.0)	0 (0.0)	0 (0.0)	0 (0.0)
103L	0 (0.0)	0 (0.0)	0 (0.0)	0 (0.0)
104R	1 (10.0)	17 (18.3)	4 (25.0)	22 (18.5)
104L	2 (20.0)	23 (24.7)	2 (12.5)	27 (29.0)
105	2 (20.0)	12 (12.9)	3 (18.8)	17 (14.3)
106recR	2 (20.0)	42 (45.2)	4 (25.0)	48 (40.3)
106recL	6 (60.0)	35 (37.6)	7 (43.8)	47 (39.5)
106pre	1 (10.0)	12 (12.9)	1 (6.3)	14 (11.8)
106tbR	0 (0.0)	0 (0.0)	0 (0.0)	0 (0.0)
106tbL	0 (0.0)	6 (6.5)	1 (6.3)	7 (5.9)
113	0 (0.0)	1 (1.1)	0 (0.0)	1 (0.8)
114	0 (0.0)	0 (0.0)	0 (0.0)	0 (0.0)

There were 70 (58.8%) patients with LNR in a single station, including 4 patients with two or more LNRs in one station. Among the other 49 (41.2%) patients, 32 (26.9%), 14 (11.8%) and 3 (2.5%) patients had LNR at two, three and four or more stations, respectively.

### Mapping of Recurrent LNs

**Figure 2:**
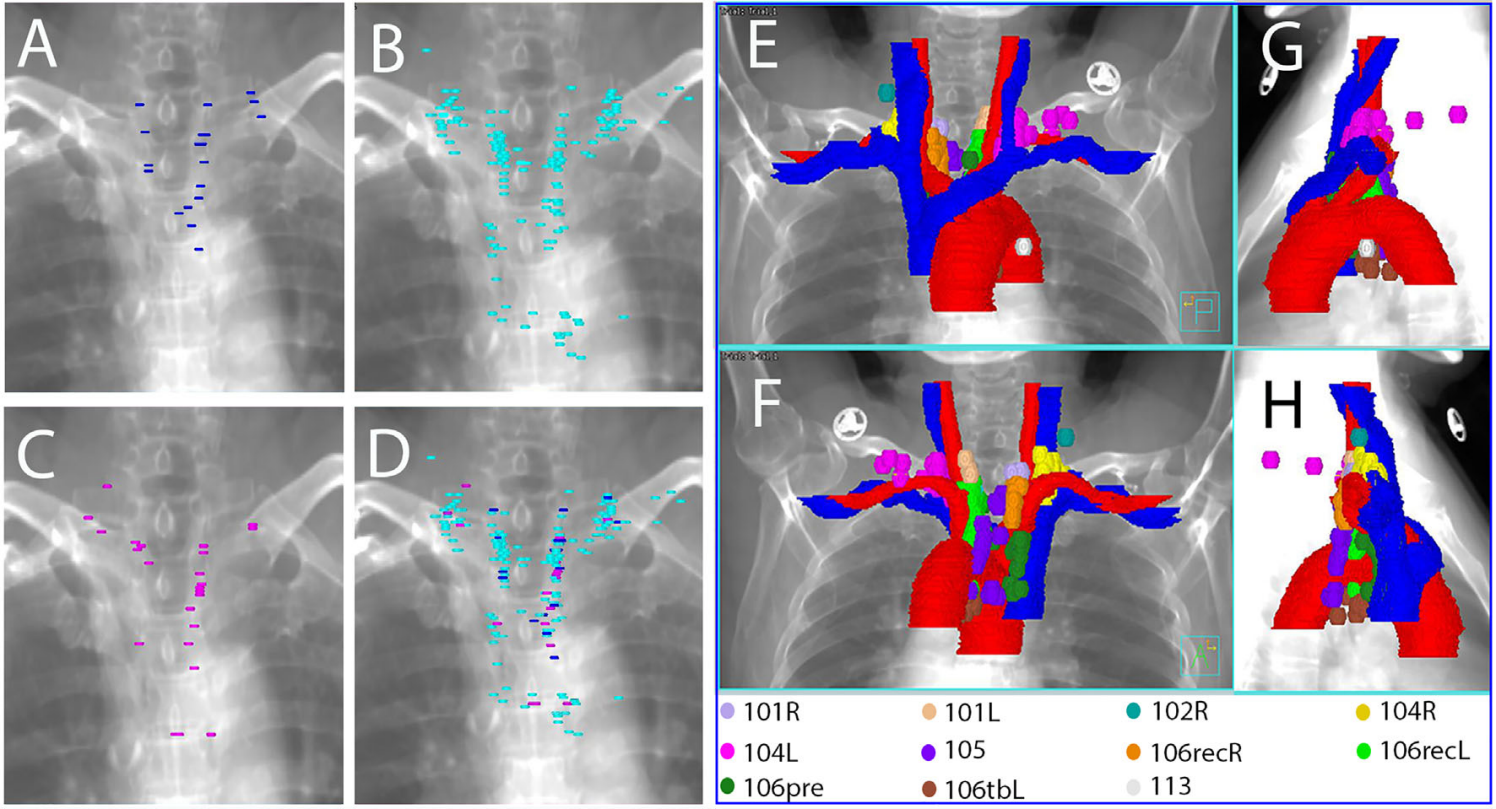
Reconstructed images with all of the plotted LNs **(A–D)** and all of the TVnds **(E–H)** on the template CT. **(A)** anterior-posterior viewpoint for upper TESCCs; **(B)** anterior-posterior viewpoint for middle TESCCs; **(C)** anterior-posterior viewpoint for lower TESCCs; **(D)** anterior-posterior viewpoint for all TESCCs; **(E)** anterior-posterior viewpoint for all TESCC; **(F)** posterior-anterior viewpoint for all TESCCs; **(G)** right-left viewpoint for all TESCCs; **(H)** left-right viewpoint for all TESCCs.

**Figure 3:**
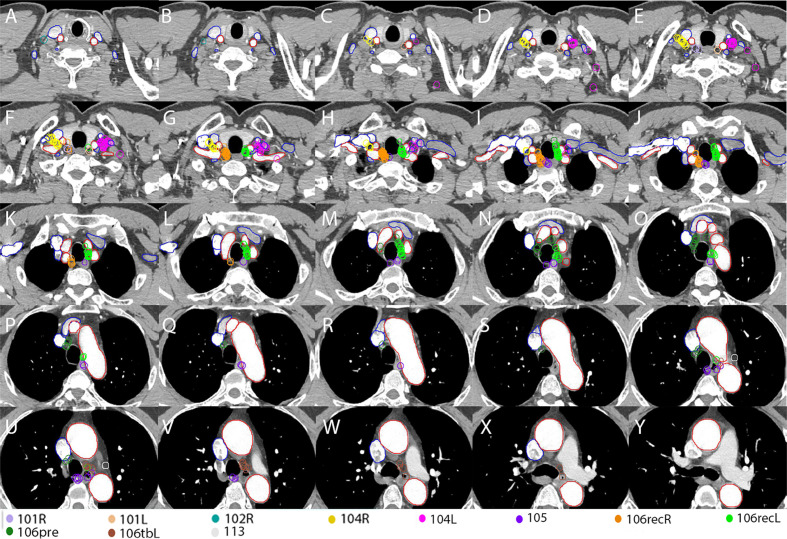
Continuous cross-sectional images (a thickness of 5.00 mm) with all of the TVnds on the template CT **(A–Y)**. Red lines: arteries; Blue lines: veins.

A total number of 215 LNs were identified. Among them, 51 (23.7%), 52 (24.2%), 26 (12.1%), 38 (17.7%), 18 (8.4%), 15 (7.0%) and 7 (3.3%) LNs were located at station 106recR, station 106recL, station 104R, station 104L, station 105, station 106pre and station 106tbL, respectively. The other 8 LNs were located in station 101R (3), station 101L (3), station 102R (1) and station 113 (1). All of the plotted LNs on the reconstructed CT images are shown in **Figures 2A–D** and all of the TVnds on the reconstructed CT images are shown in **Figures 2E–H**. The continuous cross-sectional CT images with all of the TVnds are shown in **Figure 3**.

## Discussion

The esophagus shows complex lymphatic drainage characterized by intramural longitudinal vessels and direct drainage to extramural lymphatics from the submucosal lymphatic vessels (14). As a result, lymphatic node metastasis of TEC can be found in a wide-ranging, bidirectional and skipping manner, which is challenging for designing an appropriate irradiation field for radiotherapy (15). An increasing number of studies have indicated that the CTVnd should include the lower cervical and upper mediastinal regions for PORT in TEC (5–7). However, there is no specific description of the delineation of CTVnd in these regions.

It was recommended that a modified target from the upper border of the 7th cervical vertebra to the lower border of the caudal margin of the inferior pulmonary vein could cover the high-risk regions of TESCC patients who underwent PORT (7). In our study, station 104L/R, station 106recL/R, station 105, station 106pre and station 106tbL represent the high-risk regions of LNR in TESCC. However, the frequency of LNR in these stations changed with the location of TESCC. We recommend that the CTVnd for PORT in the cervical and upper mediastinal regions cover station 101L/R, station 104L/R, station 106recL/R, station 105 and station 106pre in upper TESCC (100.0% recurrent LNs) and station 104L/R, station 106recL/R, station 105, station 106pre and station 106tbL in middle and lower TESCC (96.5% and 100.0% recurrent LNs, respectively).

The upper border of the CTVnd has usually been the cricothyroid membrane for upper TESCC and the upper margin of the first thoracic vertebra for middle or lower TESCC in many studies (16, 17). In another study, it was suggested that the target should start from the upper margin of the 7th cervical vertebra for PORT of TEC (7). In our study, we found that the upper margin should be the cricothyroid membrane because station 104L/R is a high-risk region for LNR of all TESCCs. However, station 101L/R cannot be included in CTVnd due to its low LNR for middle and lower TESCCs. Therefore, the inner upper margin is lower than the outer upper margin of CTVnd for middle and lower TESCCs (**Figure 2** and **Figures 3A–E**).

**Figure 4:**
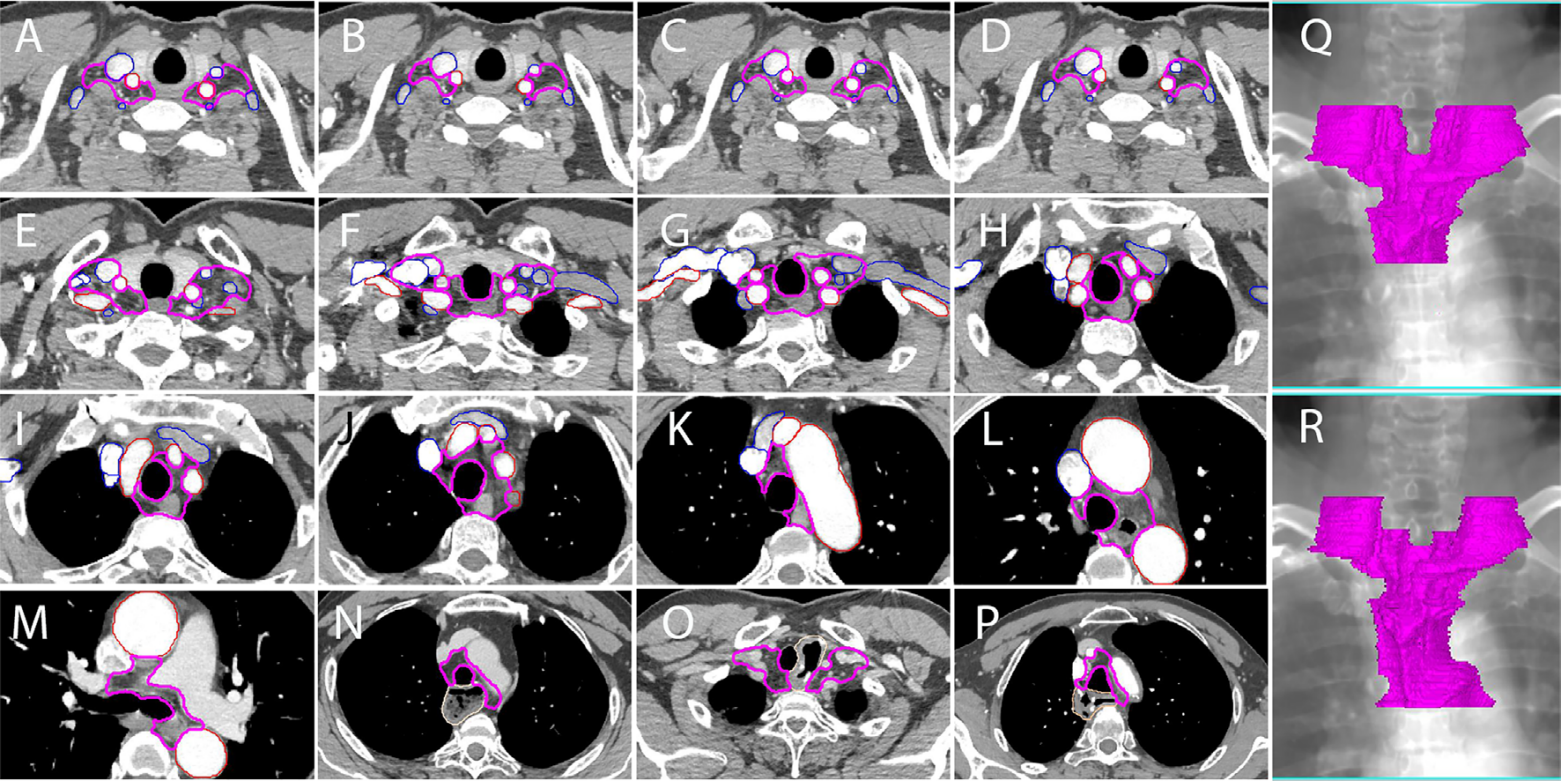
A proposed delineation of CTVnd in lower neck and upper mediastinum for PORT of TESCC. Pink lines: CTVnd; red lines: arteries; blue lines: veins. **(A, C)** at the level of the cricothyroid membrane (upper TESCC); **(B, D)** at the level of the cricothyroid membrane (middle and lower TESCCs); **(E)** at the level of the upper margin of subclavian arteries; **(F)** at the level of the lower margin of the horizonal part of subclavian arteries; **(G)** at the level of the lower margin of thyroid; **(H)** at the level of the upper margin of innominate artery; **(I)** at the level when the innominate artery is closely contact with the trachea; **(J)** at the level of the upper margin of aortic arch; **(K)** at the level when the aortic arch is closely contact with the trachea; **(L)** at the level of the upper margin of azygos vein (middle and lower TESCCs); **(M)** the level of tracheal carina (middle and lower TESCCs); **(N)** stomach in posterior mediastinum; **(O)** anastomosis in the cervicothoracic junction; **(P)** anastomosis in the level of aortic arch; **(Q)** reconstructed CTVnd on the template CT for upper TESCC; **(R)** reconstructed CTVnd on the template CT for middle and lower TESCCs.

According to the analysis of extramural lymphatic drainage of the thoracic esophagus, the lower cervical LNs were mainly located in the paratracheoesophageal area and the supraclavicular area on the outer upper sides of the venous angles (station 104L/R) (18). Similarly in other studies (10, 11), we found that the LNR at station 104L/R was located mainly on the scalene muscles in the medial groups (**Figures 3C–I**). Additionally, the lymphatics found in the medial group of station 104L/R were located on the scalenus anterior and behind the carotid sheath (19). Due to the poorly developed left paratracheal lymphatic drainage system, Virchow’s metastasis, usually on the anterior scalene muscles, contributed to higher incidence of lymph node metastasis at station 104L (14, 20). Above all, the high-risk regions for LNR in the lower neck are shown in **Figure 4A** for upper TESCC and in **Figure 4B** for middle and lower TESCCs. The LNR at station 104L/R was behind the jugular veins (**Figures 2**, **3**), confirmed by another study (11). However, these veins can be included in CTVnd in clinical practice as their filling status varied greatly. Therefore, the delineation of CTVnd in the lower neck is shown in **Figure 4C** for upper TESCC and in **Figure 4D** for middle and lower TESCCs.

Station 106recR and station 106recL are the most common regions of lymph node metastasis in TEC (14, 15, 21). In this study, station 106recR/L also had a high LNR for all TESCCs. Because vascular landmarks should be better than bony landmarks for reference in the era of conformal radiotherapy, we recommend that the inner upper border of CTVnd should be the upper margin of subclavian arteries instead of the first thoracic vertebra (**Figure 2** and **Figure 3F**) for PORT in middle and lower TESCCs. Although the superior boundary of station 105 is drawn from the cephalic border of subclavian arteries to the suprasternal notch (12), the LNR at station 105 in our study was below the horizonal part of subclavian arteries (**Figures 3H–Y**). Under the subclavian arteries, lymph vessels usually run on the subserous surface of the mediastinal pleura and connect with the lymphatics running over the arteries and scalenus anterior (19). Lymphatic vessels can run in front of or behind the right brachiocephalic vein and these vessels run in front of vertebral vein and behind the carotid sheath before joint with the right recurrent chain (station 106recR) (19). However, we found that there was no LNR in the regions on the outer and anterior sides of the brachiocephalic veins (**Figures 3G, H**). Moreover, the LNR at station 106recR was located behind the carotid sheath while LNR at station 106recL reached to the anterior carotid sheath below the thyroid (**Figures 3I, J**). Above all, the delineation of CTVnd from the upper margin of the subclavian arteries to the upper margin of innominate artery is shown in **Figures 4E–H**.

There was no LNR in the narrow space where the trachea was close in contact with the innominate artery on the right side (**Figure 3L**), the aortic arch on the left side (**Figures 3Q, S**) and the mediastinal pleura on the right posterolateral side (**Figures 3M–T**). Moreover, there was no LNR below the azygos vein (station 106tbR) and only one LNR at station 113. Therefore, the delineation of CTVnd for the rest of the upper mediastinum is shown in **Figures 4I–M**. In our study, there was no LNR in the lower part of the upper mediastinum (below the level of the cephalic border of the superior vena cava) for upper TESCC. Therefore, only part of station 106pre and station 105 should be included in CTVnd for upper TESCC. Above all, the T shaped irradiation fields are shown in **Figure 4Q** (upper TESCC) and **Figure 4R** (middle and lower TESCC). Moreover, we should attempt to avoid irradiation of the mediastinal stomach (**Figure 4N**) and make some adjustments according to the locations of anastomoses and other anatomic structures after esophagectomy in clinical practice (**Figures 4O, P**).

Compared to previous studies, our study addressed strengths as follows: 1) We admitted patients who underwent surgery alone before recurrence. The sites of LNR can most accurately represent the at-risk lymphatics for PORT; 2) Except cycles, we plotted spheres to represent the primary recurrent LNs. This mapping method was more representative and visualized on continuous cross-sectional or three-dimensional constructed CT images; and 3) Based on our results, we proposed a more reasonable atlas of CTVnd in the cervical and upper mediastinal regions, such as different recommendations of the upper border, modified ranges and boundaries at station 104, station 105, station 106rec and station 106pre, and different irradiation fields in different segments of TESCC. This detailed atlas of CTVnd from the cricothyroid membrane to the tracheal carina should be a helpful practical guide to PORT in TESCC.

There are many limitations of our study. First, it was a retrospective single-center study, and the methods of esophagectomy and lymphadenectomy were different among many patients over an 8 years period. Second, because of the strict eligibility criteria, the sample size was not sufficiently large, especially for upper and lower TESCCs. Third, it was difficult to plot all of the LNs to the primary sites on template CT images due to the great differences in anatomic structures after esophagectomy. As a result, there might be some errors in the nodal mapping. Furthermore, the optimal imaging diagnostic criteria of LNR remain controversial (22–24). More studies with large sample sizes in different centers are needed to verify our results.

In conclusion, we suggest that the CTVnd for PORT cover station 101L/R, station 104L/R, station 106recL/R, station 105 and station 106pre in upper TESCC and station 104L/R, station 106recL/R, station 105, station 106pre and station 106tbL in middle and lower TESCCs. The external group of station 104L/R and the narrow spaces where the trachea is in close contact with mediastinal structures should not be included in the CTVnd. Additionally, the lower part of the upper mediastinum (below the level of the cephalic border of the superior vena cava in our study) may not be included in CTVnd for upper TESCC. Further trials are required to evaluate the efficacy and toxicity of PORT with optimized CTVnd in the era of conformal radiotherapy.

## Data Availability Statement

The raw data supporting the conclusions of this article will be made available by the corresponding author, without undue reservation.

## Ethics Statement

The studies involving human participants were reviewed and approved by the institutional review board of the First Affiliated Hospital of Anhui Medical University. Informed consent was waived due to its retrospective design and no identifiable information was disclosed.

## Author Contributions

YW and FW conceived and designed the study. YW, DY, MK, JW and LZ contributed data collection. YW, FW, MY, XC and JJ contributed to data interpretation and statistical analysis. YW and DY prepared the manuscript. WX, NK and FW revised the manuscript. All authors contributed to the article and approved the submitted version.

## Funding

This work is supported by the Natural Science Foundation of Anhui Province (1908085QA27).

## Conflict of Interest

The authors declare that the research was conducted in the absence of any commercial or financial relationships that could be construed as a potential conflict of interest

## Abbreviations

CT, computed tomography; CTVnd, nodal clinical target volume; EC, esophageal carcinoma; LN, lymph node; LNR, lymph node recurrence; PORT, postoperative radiotherapy; TEC, thoracic esophageal carcinoma; TESCC, thoracic esophageal squamous cell carcinoma; TVnd, nodal target volume.
